# TIRADS, SRE and SWE in INDETERMINATE thyroid nodule characterization: Which has better diagnostic performance?

**DOI:** 10.1007/s11547-021-01349-5

**Published:** 2021-06-15

**Authors:** Ilaria Celletti, Daniele Fresilli, Corrado De Vito, Marco Bononi, Sara Cardaccio, Alessia Cozzolino, Cosimo Durante, Giorgio Grani, Gianmarco Grimaldi, Andrea M. Isidori, Carlo Catalano, Vito Cantisani

**Affiliations:** 1 Department of Radiological, Oncological, and Pathological Sciences, Sapienza University of Rome, Rome, Italy; 2 Department of Public Health and Infectious Diseases, Sapienza University of Rome, Rome, Italy; 3 Department of Surgery “P. Valdoni”, Sapienza University of Rome, Rome, Italy; 4 Department of Experimental Medicine, Sapienza University of Rome, Rome, Italy; 5 Department of Translational and Precision Medicine, Sapienza University of Rome, Rome, Italy; 6 Division of Radiotherapy, University of L’Aquila, L’Aquila, Italy

**Keywords:** Thyroid nodule, TIRADS, Elastosonography, Strain-ratio elastography, Shear-wave elastography

## Abstract

**Purpose:**

To assess Strain Ratio (SRE) and Shear Wave Elastography (SWE) accuracy alone and with TIRADS classification, for the risk stratification of indeterminate thyroid nodules.

**Materials and methods:**

128 Patients with 128 indeterminate nodules candidates for thyroidectomy underwent preoperative staging neck ultrasound and were classified according to K-TIRADS score. After TIRADS evaluation, semi-quantitative (SRE) and quantitative (SWE expressed in kPa) elastosonography were performed and relative diagnostic performances, alone and in combination, were compared through ROC curves analysis. In order to maximize the SRE and SWE sensitivity and specificity, their cut-off values were calculated using the Liu test. Bonferroni test was used to evaluate statistically significant differences with a *p* value < 0.05.

**Results:**

Sensitivity, specificity, PPV and NPV were, respectively, 71.4%, 82.4%, 62.5%, 87.5% for K-TIRADS baseline US, 85.7%, 94.1%, 85.7%, 94.1% for SRE and 57.1%, 79.4%, 53.3%, 81.8% for SWE (kPa expressed). SRE evaluation showed the best diagnostic accuracy compared to the SWE (kPa expressed) (*p* < 0.05) and to the K-TIRADS (*p* > 0.05). The association of SRE with conventional ultrasound with K-TIRADS score increased sensitivity (92.9% vs 71.4%) but decreased the specificity than conventional US alone (76.5% vs 82.4%).

**Conclusion:**

Strain Elastosonography can be associated with K-TIRADS US examination in the thyroid nodule characterization with indeterminate cytology; in fact, adding the SRE to K-TIRADS assessment significantly increases its sensitivity and negative predictive value. However, further multicenter studies on larger population are warranted.

## Introduction

In 2018 the "International Agency of Research on Cancer (IARC)” recorded 567,233 new cases of thyroid cancer worldwide with an incidence rate of 3.4/100,000 in men and 11.5/100,000 in women [1].

The growing incidence of thyroid neoplasms is primarily related to the "overdiagnosis" due to the increasingly widespread use of neck imaging techniques such as ultrasound; nevertheless, more than half of the newly diagnosed thyroid cancers in Italy have a low-risk of persistence or recurrence [2].

Recent papers [3] have documented the increase in incidentally thyroid lesions diagnosis that is related to neck ultrasound examinations in 67% [4], to neck CT [5], to MRI in 15% [6] and PET examinations in the remaining 1- 2% of cases [7].

Indeed, ultrasound, being a non-invasive, repeatable over time, widely diffused, and low-cost exam, allows an initial stratification of the risk of malignancy of the thyroid nodule, trying to reduce the number of unnecessary fine-needle aspiration cytology (FNAC). The risk-stratification is based on established criteria in the conventional US (B-mode) imaging: hypoechogenicity, presence of microcalcifications, the anteroposterior diameter larger than transverse diameter (taller-than-wide shape), irregular or lobulated margins [8, 9]. Several risk-stratification systems integrate all these features to group the nodules into discrete risk classes. According to the estimated malignancy risk, the clinician may decide to perform or not FNAC.

FNAC still represents the gold-standard technique for the classification of thyroid nodules, indicating the nature of the lesions with a specificity of 60–98%, but with a highly variable sensitivity (ranging between 54 and 90%) [10].

A thyroid FNAC's main problem is the frequent occurrence of an "inadequate" sampling or an "indeterminate" result.

The "indeterminate" result represents a "gray" diagnostic area, which occurs in 5–20% of cytological reports with the presence of cellular atypia of indeterminate significance (TIR3 category) [11].

TIR3 nodules' management is widely debated, considering that no more than 5–30% of them will be diagnosed as malignant at histological examination. Therefore, there is a growing need for additional diagnostic tools to define the malignancy risk of indeterminate nodules more accurately, reducing the number of surgeries for non-malignant diseases [12].

The adoption of two subcategories based on cyto-morphological features with different malignancy risk and different therapeutic indications were proposed by the Bethesda System [13], the British Thy system [14], and the Italian Consensus of Thyroid Cytology of 2014 (IRSTC: Italian Reporting System for Thyroid Cytology) [15]. In particular, the Italian classification system divided the previous indeterminate category TIR3 into two subcategories: TIR3A, identifying an indeterminate lesion with low risk of malignancy (< 10%) for which a conservative approach is suggested (ultrasound follow-up and FNAC repetition); and TIR3B, identifying a high-risk indeterminate lesion (risk of malignancy 15–30%) [12] for which surgical resection is suggested. The Italian Consensus confirmed these suggestions on the diagnosis and treatment of thyroid carcinoma [16]

The repetition of FNAC for TIR3A nodules is supported by several studies showing a benign reclassification in about 50% of cases [17]. A recent meta-analysis [18] on indeterminate nodules highlighted a malignancy rate of 17% in the TIR3A category and 52% in TIR3B [19], much higher than those expected according to the Italian Consensus of 2014. The management of each cytological class derives from a careful, combined, evaluation of clinical, ultrasound, cytological, and molecular data, if available [20–23].

Furthermore, recently, diagnostic imaging evaluation has been included additional tools such as elastosonography which evaluates the increased stiffness of thyroid nodules as a sign of malignancy [24–28]. The use of USE methods has been incorporated into international guidelines published by the World Federation for Ultrasound in Medicine and Biology (WFUMB) [29] and the European Federation of Societies for Ultrasound in Medicine and Biology (EFSUMB) [30], that also provide technical details, advantages, and limitations for strain-ratio elastography (SRE) and quantitative 2D ultrasound shear wave elastography (SWE).

The current guidelines [29–31] recommend the use of elastosonography as an integrative technique to the B-mode ultrasound evaluation, thanks to its high negative predictive value (false negatives about 3%) [31], but still no specific recommendations have been provided regarding its use for indeterminate thyroid nodules assessment.

Therefore, the present study aimed to evaluate the different diagnostic performance of the two main elastosonography techniques, semi-quantitative (SRE) and quantitative (SWE), in the risk stratification of thyroid nodules with indeterminate cytology in comparison and in addition to the B-mode ultrasound evaluation according to K-TIRADS classification (Table 1) [32].

**Table 1:** Malignancy Risk Stratification According to Korean Thyroid Imaging Reporting and Data System (K-TIRADS)

Category	US feature	Malignancy risk (%)
5 High suspicion	Solid hypoechoic nodule with any of 3 suspicious US features*	> 60
4 Intermediate suspicion	1. Solid hypoechoic nodule without any of 3 suspicious US features* or 2. Partially cystic or isohyperechoic nodule with any of 3 suspicious US features*	15–50
3 Low suspicion	Partially cystic or isohyperechoic nodule without any of 3 suspicious US features*	3–15
2 Benign	1. Spongiform 2. Partially cystic nodule with comet tail artifact 3. Pure cyst	< 3 < 1 –
1 No nodule	–	–

*Microcalcification, nonparallel orientation (taller-than-wide), spiculated/microlobulated margin

Modified from Shin et al. [32]. US = ultrasonography

## Materials and methods

### Patient enrollment

The present is a prospective study including 128 thyroid nodules in 128 patients (89 women and 39 men) with a mean age of 54.3 years (range 18–82 years) with indeterminate cytology submitted to surgery. The study enrolled patients who referred to Policlinico Umberto I from January 2017 to February 2018. All patients were euthyroid except for five patients who had subclinical hypothyroidism.

The exclusion criteria were: insufficient healthy thyroid parenchyma to perform the elastosonographic evaluation, the presence of large calcifications (> 10% of the nodule size) or large fluid areas (> 50% of the nodule size), and the unavailability of the definitive histological diagnosis (due to surgery performed elsewhere or due to patient refusal to surgery).

All patients had anesthesiology clearance for elective surgery, and acute intercurrent diseases were ruled out.

### Cytological evaluation

Ninety-five out of 128 nodules (74%) were classified as TIR3B on cytological examination and were submitted to surgery, according to the current Consensus. The remaining 33 nodules (26%) were classified as TIR3A, and they were referred to surgery for the following reasons: nodule growth during the ultrasound follow-up (9 patients); family history for thyroid neoplasms (6 patients); the concomitant presence of multinodular goiter with initial compressive symptoms (dysphonia and/or dysphagia) (8 patients); size criteria (> 4 cm) (2 patients); previous neck irradiation (2 patients); patient request, due to psychological discomfort for suspected malignancy and/or for aesthetic reasons (6 patients). In all cases, risk factors contraindicating surgery were excluded, and exhaustive and detailed counseling regarding the possible risks related to surgery was offered.

The indication for the cytological examination of TIRADS 3 nodules was based on dimensional criteria (> 1.5 cm) or other patients' risk factors (family history for thyroid cancer, previous neck irradiation, compressive symptoms, high serum calcitonin values).

### US evaluation

Patients enrolled underwent a pre-surgical ultrasound evaluation of the neck to plan the most appropriate surgical approach and redefine the site, eco structure, and size of the nodule, its loco-regional extension (with possible thyroid capsule involvement), and the involvement of the cervical lymph nodes. Moreover, each nodule underwent an elastosonographic evaluation to define the degree of hardness, expressed both in semi-quantitative and quantitative terms.

Every patient underwent the pre-surgical ultrasound and elastosonographic evaluation at least 1 month after the cytological sampling, with a mean time of 1.3 months (range 1.1–1.7 months) to avoid possible post-procedural alterations, such as hemorrhagic events, impairing nodule eco-structure and elasticity.

**Fig. 1:**
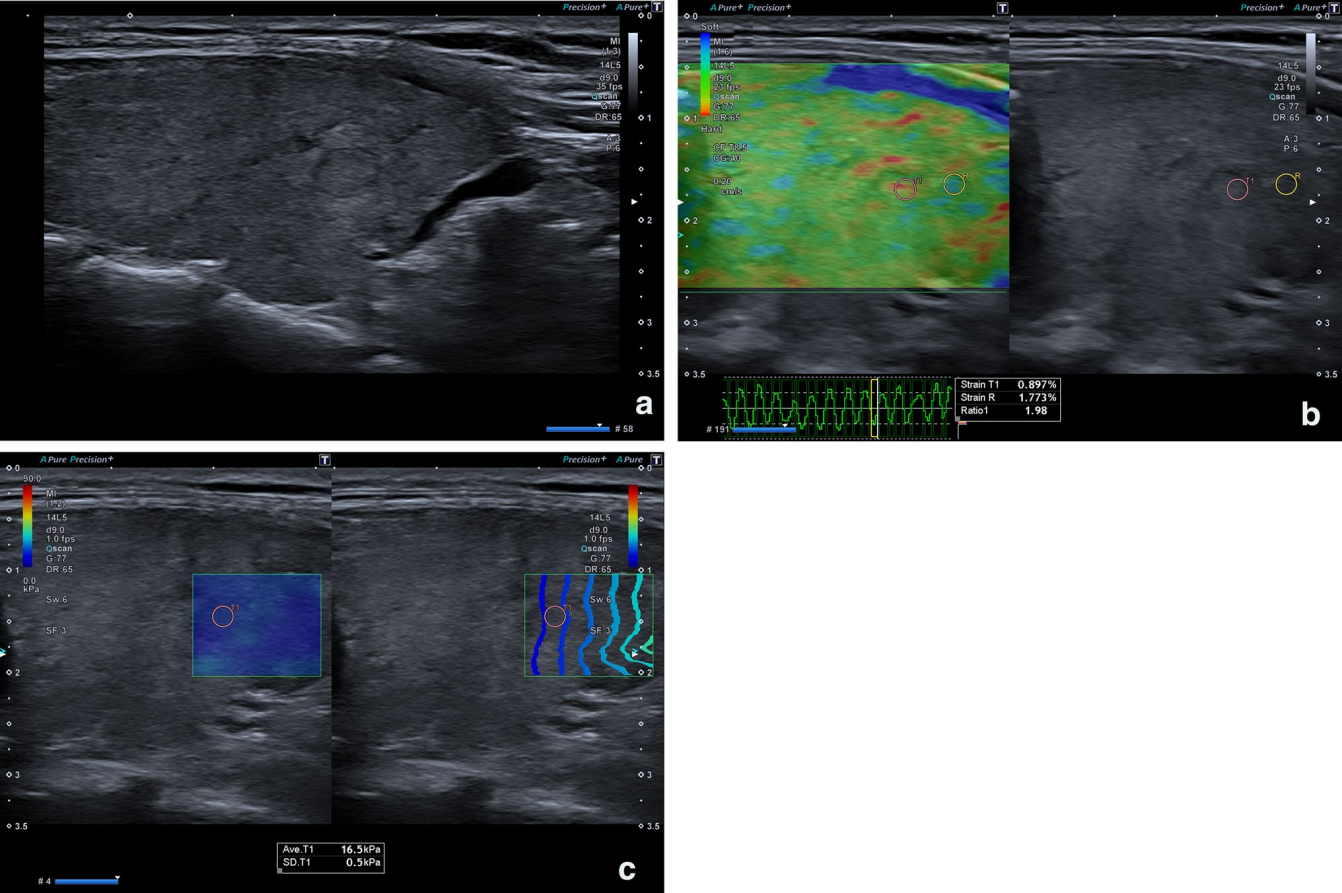
52-year-old patient with a TIR3A thyroid nodule who underwent thyroidectomy for family history for thyroid carcinoma. At histology final diagnosis was Follicular carcinoma. **a** B-mode US: Mostly hyperechoic, oval shaped, well marginated, solid thyroid nodule, with peripheral hypoechoic margin, without microcalcifications, classified as TIRADS 3; **b** Semi-quantitative Elastosonography showed a Strain Ratio value of 1.98; **c** Quantitative Elastosonography showed a value of 16.5 kPa

**Fig. 2:**
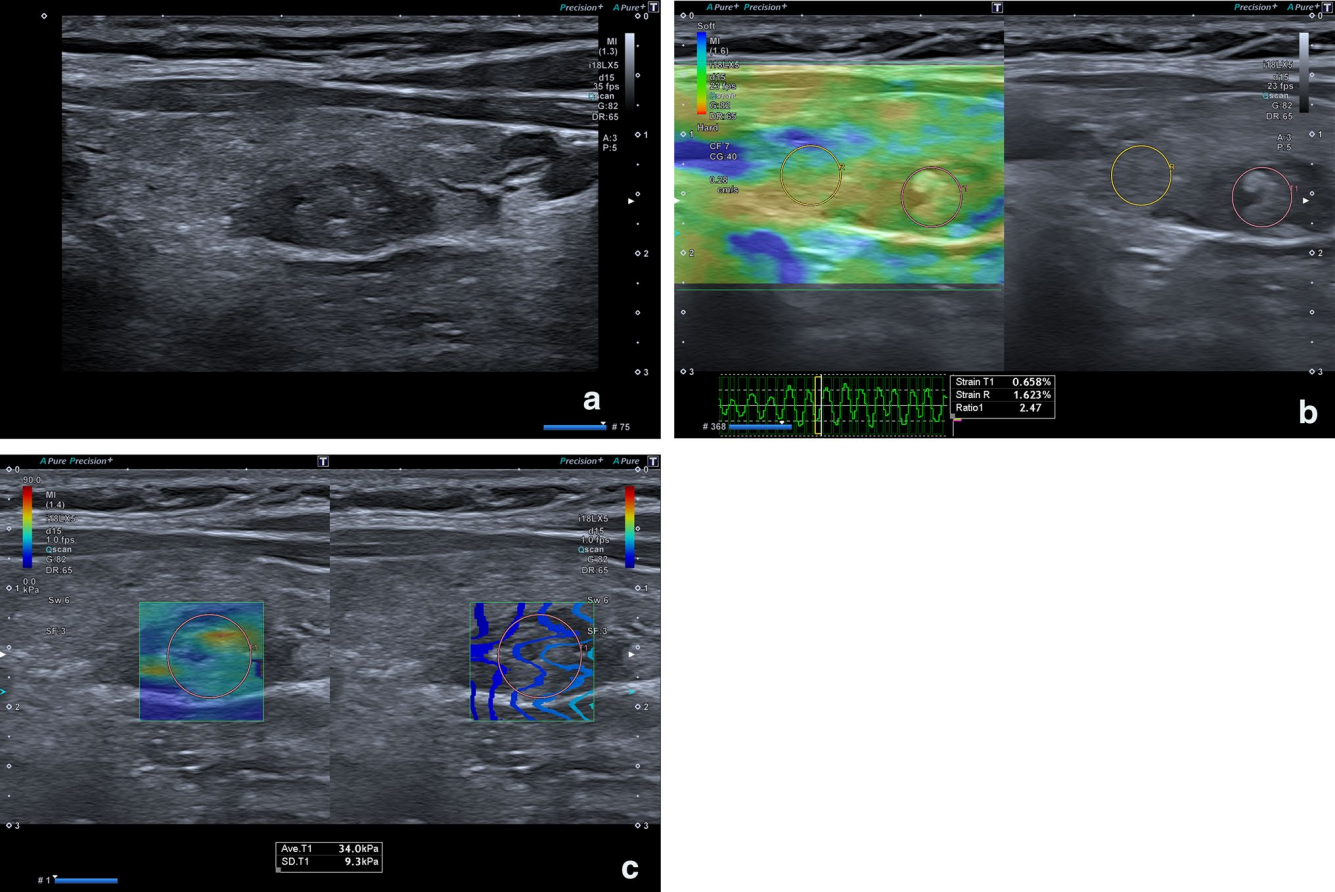
44-year-old patient with a TIR3B thyroid nodule who underwent a thyroidectomy. At histology final diagnosis was Papillary carcinoma. **a** B-mode US: Mixed, partially cystic oval shaped and lobulated thyroid nodule, without microcalcifications classified as K-TIRADS 4; **b** Semi-quantitative Elastosonography showed a Strain Ratio value of 2.47; **c** Quantitative Elastosonography showed a value of 34.0 kPa

Both the B-mode ultrasound and the elastosonographic examinations with SRE and SWE techniques were performed with Toshiba Aplio 500 or 800 (Osaka, Japan) with a linear 7–15 MHz probe (Figs. 1, 2). All nodules were evaluated by a single operator with at least 10 years of experience in elastosonography and at least 20 years in the ultrasound field within 3–15 days before surgery.

B-mode ultrasound examination was carried out following a predefined protocol aimed at describing for each nodule the following features: the eco-structure (solid or mixed), the echogenicity (hyper-, iso-, hypo-echoic, or markedly hypoechoic), the margins (regular, microlobulated or irregular), calcifications (absent, micro or macrocalcifications) and finally the shape (taller than wide or oval). In order to increase the US sensitivitiy, all nodules were attributed to a risk class following K-TIRADS score. K-TIRADS score was preferred to other TIRADS due to its greater sensitivity in identifying malignant nodules [33] in addition to easy application and high reproducibility [34] and because it is already used and well known by this same working group [35].

Therefore, it is more suited to the widespread use of TIRADS even in non-academic settings, evaluating better the role that elastosonographic techniques could have in clinical routine. On the other hand, K-TIRADS has a low specificity that has not invalidated our study because all nodules were addressed to surgery.

The ultrasound examination was completed with the elastosonographic evaluation with SRE and SWE techniques.

### SRE

Slight compressions performed SRE with the probe placed perpendicular to the skin. The examination was prospectively videotaped. The procedure was evaluated in real-time and was considered valid for subsequent evaluations after analyzing the quality indicator. Therefore, during the elastosonographic examination, both the elastogram and the reference B-mode image (twin-view) were displayed on the screen.

Once adequate compression was performed, verified by the complete and exact green color of the quality indicator box, the maximum decompression point was selected, and a ROI was manually positioned on the lesion. A second ROI, of the same size as the previous one, was in the adjacent healthy thyroid tissue at the same depth of the lesion. Then the software calculated the strain value of the tissue with semi-quantitative information (strain ratio SR).

### SWE

The quantitative elastosonographic evaluation was carried out using SWE software; the probe was kept stable perpendicular to the lesion, determining the formation of the shear-waves by a mechanical impulse, displayed in real-time to verify their correct propagation (presence of parallel waves propagated in the absence of artifacts or loss of signals). Colorimetric maps were provided, and values expressed in kPa were obtained, positioning a ROI on the thyroid lesion.

All patients underwent total thyroidectomy and the histological analysis was performed according to WHO guidelines. Post-surgical histological diagnosis was considered the gold standard.

K-TIRADS classification, SRE, and SWE values of TIR3 nodules examined and the definitive histological results were recorded in a database for statistical evaluation.

A sample size of 88 was needed to reach a 70% sensitivity and a 80% specificity with a prevalence of 30%. Width of the 95% confidence interval (95% CI) would be at most 20%.

The institutional review board approved the study, and all subjects signed informed consent.

### Statistical analysis

Data were collected in a Microsoft Excel database.

All statistical analyses were performed using Stata software (Stata version 15.0, Stata Corporation, College Station, TX, USA).

The comparison of the results was carried out with the histological examination (gold standard). The diagnostic performances of the K-TIRADS score, the SRE, and the SWE were calculated by analyzing the ROC curves. The optimal cut-off values of the continuous variables of Strain and Shear Wave were calculated with the Liu test to maximize their sensitivity and specificity, and their results were used as the basis for data dichotomization. The sensitivity, specificity, positive predictive value (PPV), negative predictive value (NPV), and area under the ROC curve (AUROC) were calculated. The comparison between the different diagnostic methods and their ROC curves was performed with the Bonferroni test, with results considered statistically significant for* p* values < 0.05.

## Results

Overall, 96 out of 128 indeterminate cytology nodules were included in the analysis (79 TIR3B and 17 TIR3A). Thirty-two nodules were excluded because of the lack of definitive histological diagnosis (28 nodules) and the presence of features not allowing a correct elastosonographic evaluation (4 nodules). In detail, these features were: coarse calcifications (*n* = 1), excessive fluid components (> 50%; *n* = 2), and incorrect compression at the SRE documented by the corresponding quality indicator (*n* = 1).

Ten patients had multinodular goiters but none of them had more than one TIR3 nodule to test.

Thirty-two out of 96 nodules were classified as TIRADS 4 and 5 at the ultrasound examination according to the K-TIRADS system, with an expected high/intermediate risk of malignancy (> 60% and between 15 and 50%, respectively). The remaining 64 nodules were classified as TIRADS 3, with an expected malignancy risk between 3 and 5%.

Final histological examination showed that 68 nodules (71%) were benign (56 follicular hyperplasia, four follicular adenomas, 6 Hürthle cell adenomas, two focal inflammatory lesions) and 28 (29%) were malignant (13 classical papillary carcinomas, 13 follicular variant papillary carcinomas, and two follicular carcinomas).

Moreover, the histological examination showed that 26 (33%) out of 79 TIR3B nodules were malignant; conversely, only 2 (12%) out of 17 TIR3A nodules were found to be malignant.

K-TIRADS assessment detected 20 true positives, 12 false positives, eight false negatives, and 56 true negatives, demonstrating a diagnostic sensitivity of 71.4%, a specificity of 82.4%, a positive predictive value of 62.5%, a negative predictive value of 87.5%, and an AUROC of 0.769.

At the elastosonographic examination, the nodules showed a mean strain ratio of 1.70 (range 0.43–4.91) and a mean SWE value of 31.97 kPa (range 0.92–90). Analyzing the ROC curves of each elastosonographic method, we calculated the optimal cut-off value discriminating the benign and malignant nature of the nodules, and the results were:

- 1.96 for the strain ratio;
- 36.80 kPa for the SWE expressed in kPa.

The semi-quantitative elastosonographic evaluation with Strain Ratio demonstrated high sensitivity (85.7%) and specificity (94.1%), identifying 24 true positives, four false positives, four false negatives, and 64 true negatives, with a positive predictive value of 85.7%, a negative predictive value of 94.1%, and an AUROC of 0.899.

The SWE expressed in kPa, showed a sensitivity of 57.1%, a specificity of 79.4%, a positive predictive value of 53.3%, a negative predictive value of 81.8%, with an AUROC of 0.683.

Overall, the SRE evaluation showed better diagnostic accuracy than SWE expressed in kPa (*p* < 0.05). The difference between K-TIRADS and SRE was not statistically significant both in terms of sensitivity and specificity.

The addition of the SRE to the SWE assessment did not show a significant increase in diagnostic accuracy. On the other hand, the addition of compressive elastosonography (SRE) to K-TIRADS assessment increased its diagnostic sensitivity (92.9% vs. 71.4%), with a slight reduction in its specificity (specificity of 76.5% vs. 82.4%). There was also an increase in the AUROC (0.847 vs. 0.769) but without statistical significance on the Bonferroni test (*p* = 0.193).

Statistical analysis stratified by sample size, revealed a reduction in the diagnostic sensitivity of K-TIRADS for nodules > 1 cm (nodule ≤ 1 cm: 85.7%; > 1 cm: 66.7%) and of SWE in kPa (nodule ≤ 1 cm: 71.4%; > 1 cm: 52.4%).

SRE showed the greatest diagnostic accuracy for nodules > 1 cm (sensitivity: 90.5%; specificity: 96.4%, PVP: 90.5%; NPV 96.4%; AUC: 0.935).

The combined evaluation of SRE with K-TIRADS determined a significant increase in diagnostic sensitivity for both nodules ≤ 1 cm (sensitivity 100% vs 85.7%) and nodules > 1 cm (sensitivity 90.5% vs 66.7%).

The SRE + K-TIRADS and SWE kPa + K-TIRADS evaluation showed the maximum diagnostic sensitivity for nodules ≤ 1 cm (100%) with a reduced specificity (SWE kPa + K-TIRADS: 50%; SRE + KTIRADS: 33.3%).

**Fig. 3:**
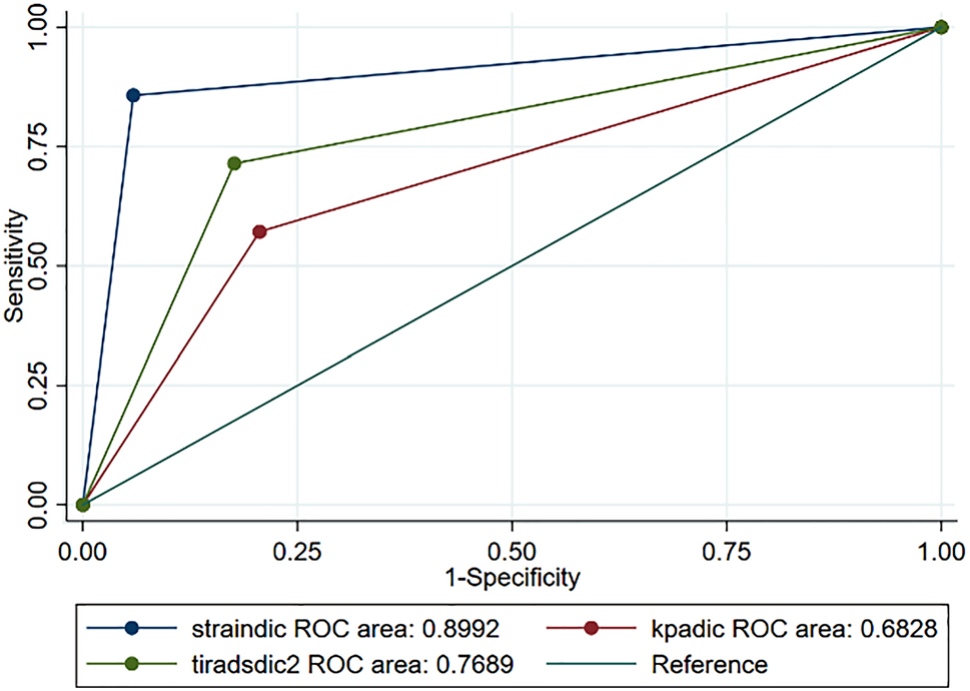
Comparison of the ROC curves for each method individually

The diagnostic accuracy of the different methods, compared individually and in combination, has been summarized in (Tables 2, 3, 4, 5 and Fig. 3).

**Table 2:**
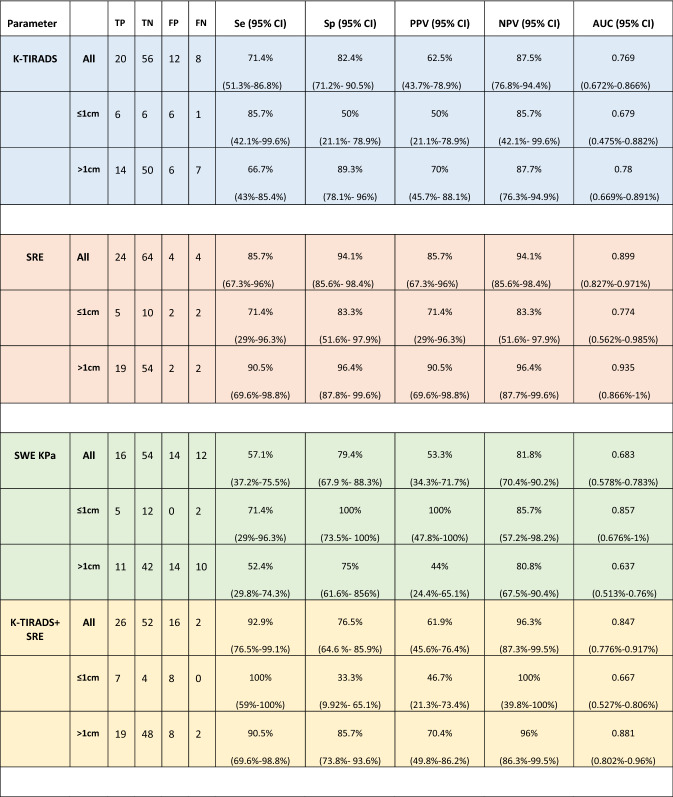
Single and multivariate analysis, stratified by size, related to the diagnostic accuracy of the different methods (K-TIRADS, SE, SWE expressed in KPa)

*K-TIRADS*: Korean Society of Thyroid Imaging Reporting and Data System; *SE*: strain elastography; *SWE*: Shear Wave elastography *TP*: true positive; *TN*: true negative; *FP*: false positive; *FN*: false negative; *Se*: sensitivity; *Sp*: specificity; *AUC*: area under the curve; *PPV*: positive predictive value; *NPV*: negative predictive value

**Table 3:** Comparison of the ROC Areas for each method individually with related Bonferroni test

	ROC area	Bonferroni
SE (standard)	0.8992	
SWE (KPa)	0.6828	0.0001
K- TIRADS	0.7689	0.1150

**Table 4:** Comparison of the combined methods (SE + K-TIRADS; SE + SWE KPa; SE + SWE Kpa + K-TIRADS) compared to the SE (standard) using the Bonferroni test

	ROC area	Bonferroni
SE (standard)	0.8992	
SE + K-TIRADS	0.8466	0.8573
SE + SWE (KPa)	0.7374	0.0000
SE + SWE (Kpa) + K-TIRADS	0.7290	0.0001

**Table 5:** Comparison between the combined methods (K-TIRADS + SE; K-TIRADS + SWE KPa) and the K-TIRADS (standard) using the Bonferroni test

	ROC area	Bonferroni
K-TIRADS (standard)	0.7689	
K-TIRADS + SE	0.8466	0.1930
K-TIRADS + SWE (Kpa)	0.7164	0.3674

## Discussion

The clinical management of thyroid nodules with indeterminate cytology still represents a highly controversial topic [36, 37].

Indeed, despite the updates of different cytological classifications, a significant number of nodules (about 20%) is still not adequately characterized on cytological examination [11, 21, 38–40] and is referred to surgery for histological diagnosis, leading to patient discomfort, possible complications and costs for the health system.

According to the most recent international guidelines (AACE-ACE-AME 2016) [36], the management of thyroid nodules with indeterminate cytology must be planned based on the cytological sub-classification, clinical data, and ultrasound features. Several potential tools are available (molecular testing [38, 41–43], core-needle biopsy, machine-learning approaches [35]), but there is no specific indication of their routine use in this category. Their cost and limited availability reduce their use in clinical practice.

Moreover, despite the availability of different imaging techniques for thyroid nodules, none of them has been proven decisive in discriminating the nature (benign or malignant) of nodules with indeterminate cytology.

Among the emerging imaging techniques in the last decade, US-elastosonography is the most promising [44]; however, its effective contribution in the clinical practice for more accurate characterization of thyroid nodules and especially for indeterminate cytopathology thyroid nodules is still not completely established.

Several studies have shown that a lower elasticity of the thyroid nodule is correlated with a higher incidence of malignancy [45, 46], although this could also be due to other histological features, such as the presence of fibrosis and the expression of galectin- 3 and fibronectin-1 [47].

A meta-analysis reported sensitivity and specificity values of 84% of 90%, respectively, for the SRE in identifying malignant thyroid nodules [48].

Another interesting study showed that the mean strain ratio found in nodules with benign histology was significantly lower than in the malignant group, indicating how the strain ratio index could help distinguish between benign and malignant nodules with a sensitivity of 98.77% and a specificity of 96.30% [49].

In 2015, Liu et al. [50] conducted a study on thyroid nodules lacking suspicious features on ultrasound examination (as in about 15% of papillary thyroid carcinomas), demonstrating the effective contribution of ARFI elastography in identifying malignant nodules, thus confirming the additional value of elastosonographic analysis to B-mode ultrasound.

The recent European (EFSUMB) [30, 31] and World (WFUMB) [29] guidelines have expressed their opinion on the new elastosonographic software, recommending the use in the evaluation of suspected thyroid nodules to be subjected to FNAC [11], thanks to their high positive predictive value (false positives about 3%) [51] and in the follow-up of patients with cytologically benign thyroid nodules. However, they have not recommended their use in evaluating indeterminate thyroid nodules since the available data are still extremely limited and often conflicting.

In this regard, a study from Cantisani et al. [52] evaluating the possible contribution of the SRE in the assessment of nodules with indeterminate cytology found a significant correlation between strain ratio values > 2.05 and malignancy risk, favoring, potentially, a pre-surgical selection with 87.5% sensitivity and 92% specificity.

Furthermore, Samir et al. [53] concluded that the quantitative approach (SWE) is a valuable tool for assessing the preoperative malignancy risk in thyroid nodules with indeterminate cytology, showing a sensitivity of 82% and a specificity of 88% with a 22.3 kPa cut-off.

Conversely, a recent study from Bardet et al. stated that the conventional SWE cut-off values could not discriminate benign and malignant tumors among thyroid nodules with indeterminate cytology [54].

However, currently, which elastosonographic technique has the greatest diagnostic accuracy is yet to be defined.

In this regard, in 2015, a meta-analysis including 54 studies on thyroid nodules (2621 malignant and 7380 benign nodules) undergoing elastosonographic evaluation with a semi-quantitative (SRE) and quantitative (SWE) technique showed that the SRE had better diagnostic sensitivity than SWE with almost overlapping specificity. Indeed, SRE showed sensitivity and specificity values of 83.0% and 81.2%, respectively, both higher than SWE (78.7% and 80.5%, respectively) [55].

More recently (2017), Hu et al. [48] published a meta-analysis including 22 studies, confirming the higher sensitivity of SRE compared to SWE (0.84 vs. 0.79) in thyroid disease, with an AUROC of 0.94 and 0.83, respectively.

The results of the few studies available on indeterminate cytology nodules seem to confirm the semi-quantitative approach (SRE) as a more performing technique than SWE in discriminating the nature of the lesions [56, 57]

In this regard, the study from Gay et al. [58], evaluating the multiparametric assessment of indeterminate thyroid nodule, concluded that the strain ratio was the only parameter significantly correlated to the histological outcomes no significant correlation of the SRE with the SWE.

Beyond the elastosonographic methods, several additional US applications were introduced in the last decade in order to improve the thyroid nodules evaluation and/or reduce its interobserver variability as CEUS and CAD systems. In a recent systematic review, CEUS showed a good sensitivity (73–93%) with a more variable specificity (63–100%) [59] Recently, US-based CAD systems for the differential diagnosis of thyroid nodules have been developed to aid the users in the image interpretation and reduce interobserver variability showing however, lesser sensitivity than expert radiologist with similar specificity [35, 60]

In our study TIR3B observed malignancy rate was slightly higher than expected by the 2014 Italian Consensus and in particular 33% vs 15–30% [16]. This is probably due to the fact that our department is considered a referral center for thyroid pathologies.

In our thyroid nodules’ cohort, we found ten multinodular goiters but none of them had more than one TIR3 nodule to test. The value of thyroid elastography for predicting nodule malignancy in nodular goiters was investigated by several authors. Different elastographic techniques were used all methods has both good sensitivity and specificity for predicting thyroid nodule malignancy [61]

The results of the current study confirm that in the diagnostic imaging algorithm aimed at defining the nature of thyroid nodules with indeterminate cytology, the semi-quantitative elastosonographic evaluation with SR shows the best diagnostic accuracy, both in terms of sensitivity (85.7%) and specificity (94.1%), compared to the SWE (*p* < 0.05) expressed in kPa (sensitivity: 57.1%; specificity: 79.4%), and to K-TIRADS (not statistically significant) (sensitivity: 71.4%; specificity: 82.4%).

Moreover, the comparison between the different methods shows that SRE evaluation integrated with TI-RADS determines an increase in diagnostic accuracy and, in particular, in its sensitivity (92.9% vs. 71.4%), reducing false negatives but with a slight increase in false positives (specificity of 76.5% vs. 82.4%).

SRE data integrated with K-TIRADS determined an increase in the NPV (NPV K-TIRADS + SRE: 96.3% versus NPV K-TIRADS 87.5%), mainly for nodules ≤ 1 cm, being both the specificity and the NPV 100%.

These results suggest that the combination of classification systems could assess indeterminate nodules malignancy risk with greater accuracy, thus reducing the number of inappropriate surgical interventions.

Therefore, strain elastography, could help to identify nodules that, although lacking suspicious ultrasound features, have a high stiffness value and thus are at risk of malignancy, for which the use of biopsy is the primary indication.

Furthermore, our results reveal how the combined use of the two elastosonographic techniques alone (SRE + SWE) and with K-TIRADS (SRE + SWE + K-TIRADS) does not determine an increase in the diagnostic accuracy of thyroid nodules with indeterminate cytology.

Our study has some limitations. First, this was a relatively small and particular cohort of thyroid nodules, all of which had already been selected for FNA biopsy and surgery by another physician (e.g., endocrinologists, oncologists, general practitioners, clinicians from other fields, pathologists), and the criteria supporting these requests were not completely known. This is reflected in the high malignancy rate. Second, this study was conducted in a single center, and all US examinations were performed by a radiologist with extensive experience in thyroid elastosonographic techniques. This may impact the applicability of our findings to other settings. Finally, our study is lacking an intra-observer and inter-observer agreement evaluation.

In conclusion, the current study results, based on the available technology, demonstrate that the compressive elastosonographic technique with SR semi-quantitative evaluation has greater diagnostic accuracy than the dynamic quantitative technique (shear-wave) in the assessment of indeterminate thyroid nodules. Therefore, it could be considered a useful tool to define the nature of thyroid nodules more accurately, especially if integrated with TIRADS.

Moreover, it would represent a powerful tool in directing patients on a personalized diagnostic and therapeutic path, thus reducing diagnostic thyroidectomies and their relevant consequences (post-procedural complications; health system costs; patient discomfort).

This is a preliminary study, and the results should be validated in a larger population, possibly in multicenter studies, to evaluate the potential bias resulting from inter-operator variability.

## Data Availability

Yes

## Abbreviations

AUC: Area Under Curve
AUROC: Area under the Receiver Operating Characteristic Curve
EFSUMB: European Federation of Ultrasound in Medicine and Biology
FNAC: Fine-needle aspiration cytology
MPUS: Multiparametric ultrasound
NPV: Negative predictive value
PPV: Positive predictive value
ROC: Receiver operating characteristic
ROI: Region Of Interest
SRE: Strain ratio elastography
SWE: Shear wave elastography
TIRADS: Thyroid Imaging, Reporting, and Data System
US: Ultrasound
WFUMB: World Federation of Ultrasound in Medicine and Biology

## Declarations

### Code availability

Strain Ratio Elastography and Shear Wave Elastography by Toshiba Aplio 500 and 800 (Osaka, Japan).
